# Genetic Architecture of a Reinforced, Postmating, Reproductive Isolation Barrier between *Neurospora* Species Indicates Evolution via Natural Selection

**DOI:** 10.1371/journal.pgen.1002204

**Published:** 2011-08-18

**Authors:** Elizabeth Turner, David J. Jacobson, John W. Taylor

**Affiliations:** Department of Plant and Microbial Biology, University of California Berkeley, Berkeley, California, United States of America; Washington University School of Medicine, United States of America

## Abstract

A role for natural selection in reinforcing premating barriers is recognized, but selection for reinforcement of postmating barriers remains controversial. Organisms lacking evolvable premating barriers can theoretically reinforce postmating isolation, but only under restrictive conditions: parental investment in hybrid progeny must inhibit subsequent reproduction, and selected postmating barriers must restore parents' capacity to reproduce successfully. We show that reinforced postmating isolation markedly increases maternal fitness in the fungus *Neurospora crassa*, and we detect the evolutionary genetic signature of natural selection by quantitative trait locus (QTL) analysis of the reinforced barrier. Hybrid progeny of *N. crassa* and *N. intermedia* are highly inviable. Fertilization by local *N. intermedia* results in early abortion of hybrid fruitbodies, and we show that abortion is adaptive because only aborted maternal colonies remain fully receptive to future reproduction. In the first QTL analysis of postmating reinforcement in microbial eukaryotes, we identify 11 loci for abortive hybrid fruitbody development, including three major QTLs that together explain 30% of trait variance. One of the major QTLs and six QTLs of lesser effect are found on the mating-type determining chromosome of *Neurospora*. Several reinforcement QTLs are flanked by genetic markers showing either segregation distortion or non-random associations with alleles at other loci in a cross between *N. crassa* of different clades, suggesting that the loci also are associated with local effects on same-species reproduction. Statistical analysis of the allelic effects distribution for abortive hybrid fruitbody development indicates its evolution occurred under positive selection. Our results strongly support a role for natural selection in the evolution of reinforced postmating isolation in *N. crassa*.

## Author Summary

Although Darwin believed that natural selection could not drive intersterility between species, it is now well established that there is a role for natural selection in the evolution of premating discrimination that reinforces barriers to hybridization. However, natural selection for postmating barriers, like hybrid inviability, is still controversial, because it can only occur when overall maternal fitness is increased by the inviability of hybrid offspring. Constraint on adaptive evolution of postmating barriers poses a problem when organisms without premating preferences must adapt to the presence of related species and ensure that reproduction occurs only between members of the same species. We studied the evolutionary genetics of a reinforced, postmating barrier between two species of mold, *Neurospora crassa* and *N. intermedia*. Although hybrids have low fitness, *Neurospora* females do not discriminate against different-species sex partners before mating. Instead, *N. crassa* has adapted to the presence of the *N. intermedia* in its range by selectively aborting hybrid fruitbodies. We show that abortion increases maternal fitness because *N. crassa* can mate again after hybridization only if fruitbodies abort. Abortion is controlled by 11 loci, whose genetic effects are consistent with an adaptive evolution model, confirming that abortion evolved via natural selection against hybridization.

## Introduction

The evolution of reproductive isolation between diverging lineages is a critical step in speciation. Most reproductive isolation barriers between taxa evolve as side effects of changes resulting from within-population processes, including, as Darwin recognized, natural selection [1]. Wallace reasoned that natural selection against maladaptive hybridization itself could drive the evolution of reproductive isolation barriers when taxa co-occur geographically (are sympatric) [2]. This mechanism, known as ‘the Wallace effect’, is also termed reinforcement, because preexisting reproductive isolation is ‘reinforced’ by natural selection for stronger barriers. Following an extensive correspondence with Wallace on this matter, Darwin remained skeptical [1]–[3]. Today, reinforcement of premating barriers by natural selection is widely accepted, but natural selection for reinforced postmating isolation still remains controversial [4]–[6].

Theoretically, reinforcement of postmating barriers can occur when 1) evolution of premating barriers is constrained, 2) there is substantial parental investment in the production and care of progeny, 3) individuals that are capable of mating more than once are unable to do so because the energetic costs of nurturing the unfit hybrids make subsequent reproduction less likely, and 4) reinforcement of the postmating barrier restores parents' capacity to successfully reproduce after hybridization [7]–[9].

After reinforcement, sympatric species or populations should show stronger barriers than those that are geographically separated (allopatric), because natural selection for stronger barriers only occurs when populations are overlapping. This biogeographic pattern has been observed for premating barriers in many animals, plants, and fungi where it has been investigated [6], [10], [11], but only a few instances of stronger postmating reinforcement in sympatry have been reported over the past 65 years [9], [12]–[14], including a microbial example involving abortion of hybrid fruitbodies (perithecia) in matings between sympatric populations of the haploid fungal species *N. crassa* and *N. intermedia* [15], [16].

### Reproductive isolation of *N. crassa* and *N. intermedia*

The geographical ranges of *N. crassa* and *N. intermedia* are broadly overlapping, and individuals of both species can be collected from the same site [15], [17], [18]. Both species are largely outbreeding, and outbreeding is confirmed by population genetic analysis [19]–[22]. Hybrids of the two species can be obtained in laboratory crosses, but natural hybrids have not been encountered [15]. This absence may reflect the rarity of hybridization in nature, the low viability of hybrids, or both. Nevertheless, phylogenetic conflict between some gene trees and the species tree of the *Neurospora* genus indicates historical hybridization and introgression between *N. crassa* and *N. intermedia* [23].

In *Neurospora*, mating can occur only between individuals having different alleles at the mating-type locus (*mat a* or *mat A*). Under nutrient limited conditions, a haploid colony of *Neurospora* differentiates female reproductive structures (protoperithecia). Fertilization occurs when a specialized hypha (trichogyne) growing from a protoperithecium fuses with a cell from a colony of the opposite mating type. The attraction of trichogynes to fertilizing cells is mediated by mating-type specific pheromomones. After fertilization, nuclei from the fertilizing strain travel through the trichogyne to the protoperithecium, where karyogamy eventually occurs. A series of independent meiotic events give rise to the sexual progeny (ascospores), which develop within flask shaped fruitbodies (perithecia) on the maternal thallus. Upon maturity, the ascospores are forcibly ejected from the fruitbody.

In *Neurospora*, evolution of premating isolation is apparently constrained because the sequences of the mating-type–specific, peptide pheromones controlling attraction between trichogynes and fertilizing cells are conserved throughout the genus (as determined by BLAST analysis of the *N. crassa*, *N. tetrasperma*, and *N. discreta* genomes [24]) and even beyond [25]. Evolution of the extracellular, ligand-binding portions of mating-pheromone receptor proteins is also comparatively constrained [26]. In *Neurospora*, sex cells of mating-type–compatible partners usually fuse before incompatibilities are expressed, and incompatibility arises either prezygotically in the fusion cell and the subsequent dikaryotic cells that proliferate from it, or postzygotically during the meiosis that directly follows karyogamy and during the formation and development of the ascospores [17]. Since *Neurospora* progeny develop within fruitbodies composed entirely of maternal tissue, the maternal colony (mycelium) bears virtually the entire cost of reproduction. Because 98% of *N. crassa*×*N. intermedia* hybrid progeny are inviable [15], and because allocation of resources to developing fruitbodies on one part of the colony abolishes the fertility of uncrossed regions of the colony [27], hybridization is severely maladaptive.

The key questions are: 1) Does abortion of hybrid fruitbodies by *N. crassa* make subsequent reproduction possible for the maternal colony, thereby conferring a fitness advantage? and 2) Did this postmating barrier evolved by natural selection? First we show that hybrid fruitbody abortion is adaptive, because it preserves the fertility of maternal *N. crassa*. Then by quantitative trait locus (QTL) analysis of hybrid fruitbody abortion and statistical analysis of the allelic effects distribution for the detected loci, we show that the genetic architecture that we observe is consistent with evolution by positive natural selection.

## Results

### Abortion of hybrid fruitbodies is adaptive

We tested whether female fertility of colonies was preserved after abortion of sympatric hybrid fruitbodies in sequential mating experiments between the two Neurospora species. *N. crassa* colonies were grown in Petri plates on synthetic cross agar medium, which promotes sexual reproduction. One half of the receptive *N. crassa* colony was fertilized by either an allopatric or sympatric *N. intermedia* strain, and the effect of allopatric vs. sympatric fertilization on the reproductive success of subsequent conspecific fertilization on another portion of the maternal colony was assayed. Initial fertilizations by a conspecific strain or with water (pseudo-fertilizations) were also performed as controls. Each of the four initial-fertilization treatments (allopatric male, sympatric male, conspecific male, pseudo-fertilization) was replicated three times. Following Dettman, et al., reproductive success was scored on a seven-category scale incorporating fruitbody development and quality of ejected ascospores, if any [15]. The *N. crassa* and *N. intermedia* strains used are described in the Materials and Methods and listed in Table 1. In all sequential matings, both the maternal strain and conspecific fertilizing strains were *N. crassa* from the NcC clade endemic to India, hereafter referred to as NcC-India [15].

**Table 1:** Strains of *Neurospora* used in this study.

Strains		Mating type	Species^3^	Geographic location	
FGSC^1^	D^2^			Region^4^	Collection Site
8903*	143	A	*N. crassa* (NcA)	Carib. Basin	Marrero, Louisiana
8865†	105	A	*N. crassa* (NcC)	India	Madaurai, Tamil Nadu
8866* †	106	a	*N. crassa* (NcC)	India	Rameshwaram, Tamil Nadu
8833‡	73	a	*N. intermedia*	Africa	Adiopodoume, Ivory Coast
8843†	83	A	*N. intermedia*	Africa	Makokou, Gabon
8825† ‡	65	A	*N. intermedia*	Africa	Yopougon, Ivory Coast
8824†	64	A	*N. intermedia*	Carib. Basin	Carrefour Mme. Gras, Haiti
8786†	26	A	*N. intermedia*	Carib. Basin	Homestead, Florida
8869‡	109	a	*N. intermedia*	India	Madaurai, Tamil Nadu
8861†	101	A	*N. intermedia*	India	Mallilinatham, Tamil Nadu
8808† ‡	48	A	*N. intermedia*	India	Rameshwaram, Tamil Nadu

*Neurospora* strains were used to study the genetics and evolution of postmating reproductive isolation between sympatric populations of *N. crassa* and *N. intermedia*.

1 FGSC strain numbers are from the Fungal Genetics Stock Center. Symbols after strain numbers are as follows:

*Parent of the *N. crassa* NcC × NcA QTL mapping population;

**†:** Strain used in sequential mating experiments;

**‡:** *N. intermedia* strain crossed to the QTL mapping population.

2 D numbers are as assigned in [15].

3 As determined in [15], with infraspecific subgroups in parentheses.

4 Carib. Basin, Caribbean Basin, which includes the coastal areas along the Gulf of Mexico and Caribbean Sea and the islands within. East Asia includes east of India and the Pacific Islands.

**Figure 1:**
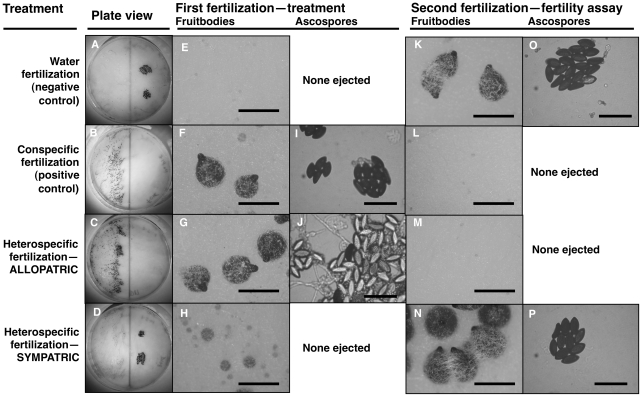
Maternal fitness of sequentially mated *Neurospora*. To determine the effect of hybrid matings on subsequent conspecific matings, receptive N. crass NcC-India cultures were initially fertilized in four different ways: distilled water as a negative control (A, E, K, O); *N. crassa* NcC-India as a conspecific positive control (B, F, I, L); *N. intermedia* allopatric to *N. crassa* NcC-India (C, G, J, M); *N. intermedia* sympatric to *N. crassa* NcC-India (D, H, N, P). In all experiments, the second fertilizing strain was an *N. crassa* NcC-India. The photographs show: whole plates (A–D), typical fruitbody development (E–H, K–N; bar = 500 µm) and ejected ascospores, if any (I, J, O, P; bar = 50 µm), resulting from the first and second fertilizations. Note that the conspecific second fertilizations resulted in ascospore production only when the initial heterospecific partner was a sympatric strain or when the initial fertilization was a water control (P and O, respectively). Second-fertilization sexual development was completely inhibited after initial fertilization by allopatric heterospecifics or by the conspecific postitive control (M and L, respectively). The clear ascospores in J are inviable hybrid progeny typical of crosses between allopatric strains of *N. crassa* and *N. intermedia*.

Fruitbody development on portions of the maternal colony fertilized by allopatric *N. intermedia* strains is normal and results in ascospore ejection, although a majority of the hybrid ascospores are unmelanized and inviable (reproductive success score (RSS) = 4.33±0.14, Figure 1). Following allopatric hybridization, response to conspecific fertilization at the second time point is completely inhibited (RSS = 0.00±0.00).

In contrast, fertilization by sympatric *N. intermedia* strains at the first time point yields only aborted fruitbodies (RSS = 1.00±0.00), but the subsequent conspecific matings are fully fertile (RSS = 6.00±0.00, Table 2). We performed semi-parametric regression using a proportional hazards survival model [28] to examine the effects of fertilization at the first time point (water control, conspecific control, or allopatric or sympatric heterospecific) on progression through the sexual cycle after fertilization at the second time point (measured as RSS). The first–time-point fertilization treatment had a significant effect (P<0.0028), whereas the nested effects of geographic origin and strain identity of the first–time-point male do not have significant effects (Table 3). We conclude that abortion of hybrid fruitbodies is selectively advantageous because abortion preserves maternal fertility of the colony after hybridization.

**Table 2:** The effect of initial fertilization on subsequent maternal fertility in sequentially fertilized *Neurospora crassa* (NcC-India) colonies.

Species of initial fertilizing male (conspecific, allopatric or sympatric)	Strain (n)	Region	RSS^1^ of initial fertilization mean (SE)	RSS of subsequent fertilization mean (SE)
Water control	na (n = 3)	na	0.00 (0.00)	6.00 (0.00)
*N. crassa* (conspecific)	8865 (n = 3)	India	6.00 (0.00)	0.00 (0.00)
*N. intermedia* (allopatric)	8786 (n = 3)	Carib.^2^	5.00 (0.00)	0.00 (0.00)
*N. intermedia* (allopatric)	8824 (n = 3)	Carib.	4.00 (0.00)	0.00 (0.00)
*N. intermedia* (allopatric)	8843 (n = 3)	Africa	4.00 (0.00)	0.00 (0.00)
*N. intermedia* (allopatric)	8825 (n = 3)	Africa	4.33 (0.33)	0.00 (0.00)
*N. intermedia* (sympatric)	8808 (n = 3)	India	1.00 (0.00)	6.00 (0.00)
*N. intermedia* (sympatric)	8861 (n = 3)	India	1.00 (0.00)	6.00 (0.00)

One half of a *Neurospora crassa* (NcC-India) colony was fertilized by a conspecific strain or by *N. intermedia* from sympatric of allopatric populations (initial fertilization). Five days later, the other half of the colony was fertilized by a conspecific strain (subsequent fertilization). The experiment shows the reproductive success of initial fertilizations and subsequent fertilizations.

1 Carib. = Caribbean Basin.

2 RSS = Reproductive success score, based on a seven-stage scale where 0 represents no sexual response to fertilization, and 6 represents normal fertility.

**Table 3:** Proportional hazards model of how initial fertilization affects subsequent maternal fertility of *Neurospora* colonies.

Test	Source	−Log Likelihood	DF	Chi Square	Prob>Chi Square
Whole model^1^					
	Difference	7.05	7	14.10	0.0494
	Full	60.40			
	Reduced	67.45			
Effects					
	Type of first fertilization		3	14.10	0.0028
	Geographic origin of first fertilizing strain		1	0	1.0000
	First fertilizing strain identity		3	0	1.0000

The effects of the type of first fertilization (pseudo-fertilization, conspecific, allopatric heterospecific, sympatric heterospecific) and the geographic origin and strain identity of the initial fertilizing strain on subsequent fertility of the maternal colony were assessed by a proportional hazards model.

1 The model incorporates the type of initial fertilization (water negative control, conspecific, allopatric heterospecific, or sympatric heterospecific), the geographic origin of the initial fertilizing strain, and the strain identity of the initial fertilizing strain as nested effects.

### Design of QTL mapping experiments

Previous research on the genetics of reinforcement focused on premating barriers in animals [29]–[31]. Here we investigate whether the genetic architecture of a reinforced, microbial, postmating barrier is consistent with evolution by directional natural selection [32], [33]. A 500-member, *N. crassa* mapping population was derived from an intraspecific, inter-clade cross between the NcC-India strain FGSC 8866, and a Louisiana, USA, strain, FGSC 8903, a member of the NcA clade, hereafter referred to as NcA-Louisiana. *Neurospora* are hermaphroditic so that the parental strains could be mated reciprocally. Based on the identity of the maternal parent we can infer that the mapping population contains both individuals with NcA-Louisiana cytoplasm (57%) and individuals with NcC-India cytoplasm (43%).

The *N. crassa* mapping strains were crossed maternally and paternally to *N. intermedia* strains from Tamil Nadu, India, which are sympatric to the NcC-India parent and allopatric to the NcA-Louisiana parent. NcC-India aborts fruitbodies after fertilization by *N. intermedia* from India, but NcA-Louisiana does not [15], [16]. The mapping strains were also crossed maternally and paternally to African *N. intermedia* strains, which are allopatric to both the NcC-India and NcA-Louisiana parents. Neither the NcA-Louisiana parent nor the NcC-India parent aborts fruitbodies after fertilization by the African *N. intermedia* strain. The four traits that we studied were fruitbody development in the four different types of matings. The four types of matings are defined by the parental role of the mapping strain (maternal or paternal) and the geographic relationship of the *N. intermedia* strain (Indian and therefore sympatric to the NcC-India parent of the mapping population, hereafter termed “sympatric”; or African, and therefore allopatric to both the NcC-India parent and the NcA-Louisiana parent, hereafter termed “allopatric”; see Table 4). Therefore each member of the mapping population was crossed twice to an Indian *N.* intermedia strain and twice to an African *N. intermedia* strain, once with the mapping strain in the maternal role and once with the mapping strain in the paternal role, for a total of four crosses per mapping strain. We examined fruitbody development in each cross, recording its fruitbody development score (FDS) 10 days after fertilization (see Materials and Methods).

**Table 4:** Summary of hybrid fruitbody development phenotypes analyzed by QTL mapping.

Trait symbol	Role of *N. crassa* mapping strains^1^	Origin of *N. intermedia* tester strains	Sympatric or allopatric^2^
A	Maternal	India	Sympatric
B	Paternal	India	Sympatric
C	Maternal	Ivory Coast	Allopatric
D	Paternal	Ivory Coast	Allopatric

The *N. crassa* QTL mapping strains were crossed reciprocally to *N. intermedia* tester strains from India and Africa. Hybrid fruitbody development was measured in each of these four types of crosses.

1 The *N. crassa* mapping strains are derived from an inter-clade NcA-Louisiana × NcC-India f_1_ cross.

2 Crosses to Indian tester strains, which are sympatric to the NcC-India parent of the mapping population, are designated sympatric. African tester strains are allopatric to both parents.

### Linkage and segregation analysis

**Figure 2:**
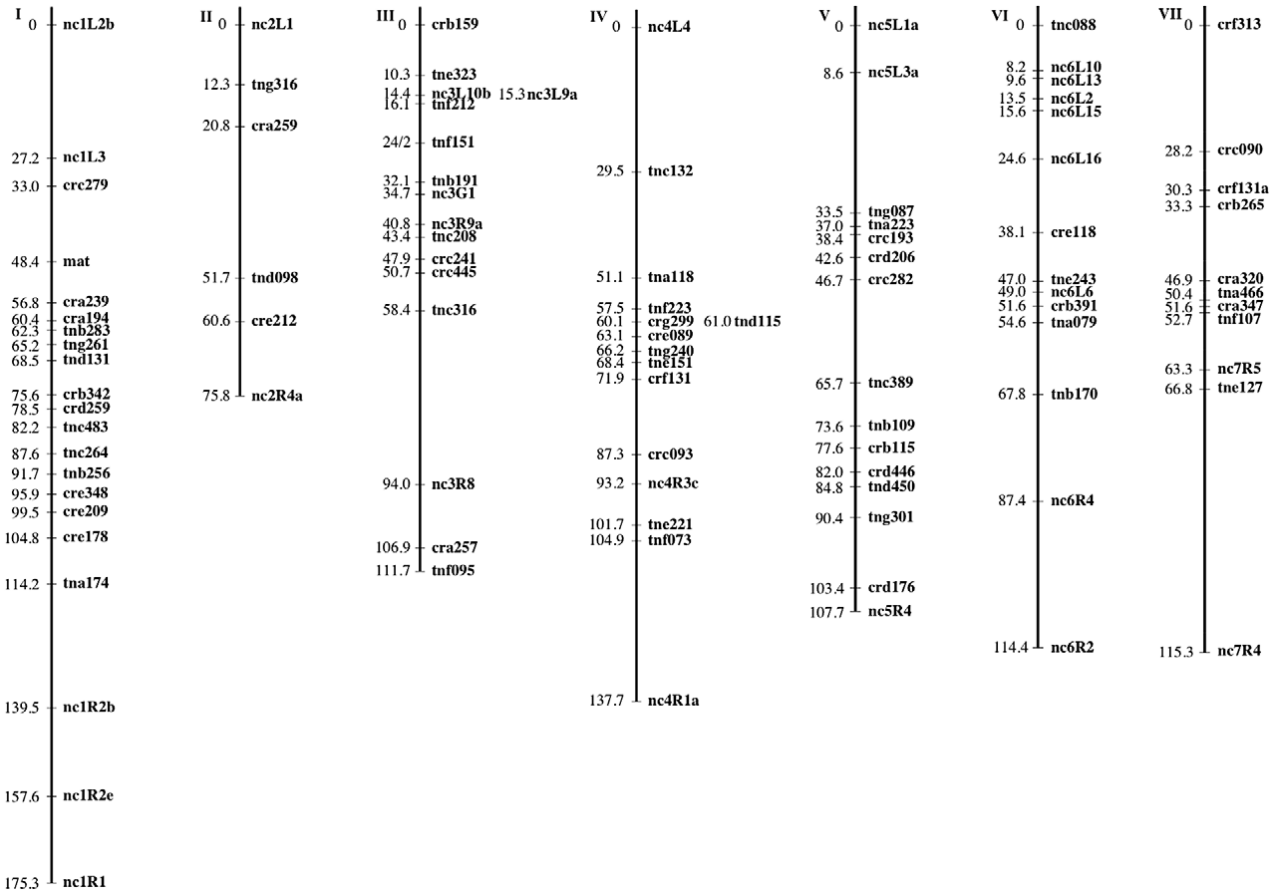
The *N. crassa* clade NcA × clade NcC genetic map. Markers are mapped to seven linkage groups reflecting the seven chromosomes of *N. crassa*. For each marker, its genetic position (cM) relative to the linkage group's leftmost marker is given. Marker names indicate the nature of each marker as follows: AFLP markers are named with a three-letter prefix and the estimated length of the fragment in base pairs. The prefixes “cr” and “tn” indicate whether the AFLP fragment was present in the NcA or NcC parent, respectively. The third letter in the prefix (a–h) indicates which selective primers were used to obtain the fragment. Microsatellite markers are named with the prefix “nc” followed by the number of the linkage group and (1–7) and an alphanumeric identifier preceded by “L” or “R” to reflect whether the microsatellite was targeted to the left arm or the right arm of the chromosome.

The mapping strains were genotyped at 69 AFLP (Table 5 and Table 6) and 28 microsatellite (Table 7) markers as well as the *mat* locus. A genetic map containing seven linkage groups (LG) reflecting the seven chromosomes of *Neurospora* was estimated, with a total map length of 837.9 cM and an average intermarker distance of 9.2 cM (Figure 2).

**Table 5:** Primer sequences for preselective and selective AFLP primers.

Primer^1^	Sequence (5′ to 3′)
E-00	GACTGCGTACCAATTC
E-AA	GACTGCGTACCAATTCAA
E-TA	GACTGCGTACCAATTCTA
E-TC	GACTGCGTACCAATTCTC
M-00	GATGAGTCCTGAGTAA
M-AA	GATGAGTCCTGAGTAAAA
M-AC	GATGAGTCCTGAGTAAAC
M-AG	GATGAGTCCTGAGTAAAG
M-CA	GATGAGTCCTGAGTAACA
M-CG	GATGAGTCCTGAGTAACG
M-GC	GATGAGTCCTGAGTAAGC

Primer sequences for the AFLP markers incorporated into the *N. crassa* linkage map are listed.

1 E- and M- indicate whether the primer was designed to anneal to the EcoRI or the MseI restriction-site adaptor, respectively. Character pairs following the restriction enzyme designation indicate that the primer is preselective (00) or shows the two additional 3′ nucleotides added to selective primers.

**Table 6:** Primer pairs for preselective and selective AFLP reactions.

E-primer	M-primer	Primer pair type	Letter code^1^
E-00	M-00	preselective	—
E-AA	M-AC	selective	a
E-AA	M-AA	selective	b
E-AA	M-AG	selective	c
E-TA	M-CG	selective	d
E-TA	M-CA	selective	e
E-TC	M-CG	selective	f
E-TC	M-GC	selective	g

The AFLP markers incorporated into the *N. crassa* linkage map were obtained with seven different primer-pair combinations. The preselective primer pair used was the same in each case.

1 In the names of mapped AFLP markers this letter code is the third letter of the three-letter prefix.

**Table 7:** Microsatellite targeted markers on the *Neurospora crassa* linkage map.

Marker^1^	Position (bp)^2^	μsat^3^	Fragment size (bp)		Primers (5′ to 3′)	
			NcA	NcC	Forward	Reverse
nc1L2b	293960 +	12 aga	103	170	tacattcccctggactcctgg	gtttcttcgggcgttgag
nc1L3	788170 +	10 tgt	177	163	gggaacacaaagaacgaaaga	cgatacgatacgatgcgatac
nc1R2b	9103466 +	17 caa	276	313	cgttcttcttcttccgcttg	aggttcagggtgctcgtct
nc1R2e	9208198 +	12 ttc	245	259	cgcctgctgaataaagaact	tcacactcaccctcctcctc
nc1R1	9646074 −	11 gct	282	263	gagcagtcccagaagaccag	cccttcctgcaacgtattgt
nc2L1	59713 −	8 aag	414	404	ggaagaaaggaatgggtggt	ccaggtgttcaatgcatgtc
nc2R4a	3472577 +	12 ctt	192	178	cccattactcctgaacaagca	tccacctcattttcctcacc
nc3L10b	1222692 −	8 gaa	210	198	ggtccgtgggctgtatctt	gtggaagggggtttagagga
nc3L9a	1411723 +	12 caa	456	455	gccgagttaggtcttcagga	agggtgaggggttggtagag
nc3R1	2989674 +	19 gac	216	182	aggactcggacgatgagaga	atccccatctaccccttcac
nc3R9a	3754818 +	41 tgt	401	466	gcatacacgggctcttccta	tcacctcctcatacctcttca
nc3R8	4883113 +	9 gat	226	233	ggacttttgggcgggtag	gcgaatggaggagggttg
nc4L4	58410 −	16 aca	231	219	tcttggctgtgatgttgctc	gtcaccgtctgtcgtctcct
nc4R3c	4951719 +	14 tgt	201	194	ggcggttggtaggaaatgtt	tgctcggttgataggattcg
nc4R1a	5905767 −	15 aca	124	95	gggggaaacaactacactcttt	aatgctgatgacgatgatgg
nc5L1a	113742 −	9 ctt	301	308	ggagtggtcctttgtagagtcg	ccctcagttcccatcaaga
nc5L3a	363346 −	12 caa	373	411	gccctgctttgttcatctgt	acttcacctgcttgcttgct
nc5R4	5641416 −	9 tct	265	267	gtggtggtggttcaggttgt	tggcttcagttctttctttcc
nc6L10	302027 +	8 gat	201	195	ttgggtgcatacatcatcgt	cattggtgacggctttctct
nc6L13	350117 +	7 tct	229	224	actcggaattttgcctgttg	agttaaaactgccgggaagg
nc6L2	425652 +	7 ggt	318	306	tggcgaggtctttgagttct	aagacgagaccgggattctt
nc6L15	495534 +	12 gaa	169	143	tccgaataaggaaagtaggtagtc	cttcagggtccgagacagag
nc6L16	631408 +	8 tcc	213	210	tcggaaagaaagagagagtgaaa	atgaagatgctgggaggaga
nc6L6	1568043 +	8 ctg	326	332	gacgagaaagcggagaaac	tgggcgggtggattagtg
nc6R4	3762945 +	16 gaa	183	153	tttgtccagagccagatgc	tctccgaaaatgtccaaca
nc6R2	4094250 +	24 cat	194	144	gggagttgagtgtcgagagc	agagctgggaatatccacga
nc7R5	3383817 −	14 agg	240	198	tgttgttcgtatgggtttgg	cgtggtgtgtatcgctttca
nc7R4	4190212 −	16 caa	220	180	ggtagaagacgaagccgaag	agcaccaccaagagttcacc

The linkage group, chromosome position, repeat, number of repeats, allele size, and primer sequences are given for all of the microsatellite markers incorporated into the *N. crassa* linkage map.

1 Each microsatellite marker name consist of the prefix ‘nc’ followed by a numeral indicating linkage group, then the letter ‘R’ or ‘L’ indicating right or left arm of the chromosome, and then an alpha-numeric identifier.

2 Supercontigs refer to assembly 10 of the *Neurospora crassa* genome by the Broad Institute. The position of the targeted repeat is given.

3 μsat is microsatellite.

**Figure 3:**
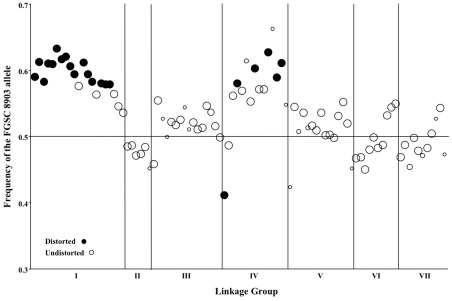
Segregation of mapped markers in the *N. crassa* NcA × NcC mapping population. Genetic markers showing distorted (closed circles) or undistorted (open circles) segregation are ordered according to their position within the seven linkage groups on the x-axis. The size of each circle is proportional to the number of individuals in the mapping population that was genotyped for that marker, and the frequency of the NcA allele is shown on the y-axis. Four markers from linkage group VI (nc6L2, nc6L6, nc6L13, and nc6L15) were excluded because they were selectively genotyped for finer mapping.

Non-Mendelian segregation and non-random associations (NRA) among alleles at multiple loci can reflect genetic incompatibilities between the NcC and NcA genomes. Individuals in the mapping population inherited 53.6% of genotyped alleles from the NcA-Louisiana parent, and this is significantly higher than the 50% expected under Mendelian segregation (t Test, *P*_t = 6.8721, DF = 93_<0.0001). The proportion of NcA-Louisiana alleles inherited varied across linkage groups (ANOVA, *P*_F ratio = 23.34, DF = 93_<0.0001), with linkage groups II, VI, and VII showing below 50% inheritance of NcA-Louisiana alleles (Figure 3). The skew towards NcA alleles was strongest on linkage group I, with 59.0% of marker alleles inherited from that parent. Of 22 markers showing significant segregation distortion (χ^2^, *P*<0.05), only one marker on LG IV (nc4L4) was significantly skewed in favor of the NcC-India allele, while the 21 other significantly distorted markers favored the NcA-Louisiana and were located on LG I (16 markers) and LG IV (5 markers).

Seven marker pairs representing four pairs of linkage groups exhibit significant non-random associations (Table 8). Positive non-random associations, reflecting an overrepresentation of parental haplotypes, are consistent with the existence of negative epistasis among clade specific alleles, as predicted by the Dobzhansky-Muller (D-M) model of the evolution of genetic incompatibilities first articulated by Bateson [34]. Per this model, incompatibilities between two loci, X and Y, must arise in in a two-step fashion, as follows: Consider that at the time that the NcA and NcC clades diverged, both populations contained ancestral (anc) alleles at every locus. The first fixation of a derived allele, e.g., X_anc_→X_NcA_, will not cause incompatibility, because X_NcA_ arises in an ancestral genetic background and must also be compatible with the ancestral alleles that comprise the NcC genome. However the next derived allele to be fixed by either population can be a source of incompatibility because the genetic backgrounds of the two clades are no longer identical. For example, if we consider fixation of a new Y allele in the NcC population, Y_anc_→Y_NcC_, we see that Y_NcC_ evolves in the presence of X_anc_, and may not be compatible with the previously derived allele X_NcA_ in the NcA clade. Conversely, fixation of a new Y allele in the NcA population, Y_anc_→Y_NcA_ can also give rise to incompatibilities, since Y_NcA_ need not be compatible with the X_anc_ allele, which is still fixed in the NcC population.”

**Table 8:** Pairs of unlinked loci showing significant non-random associations.

LG	Markers	Haplotype counts^1^ (observed/expected^2^)				D′^3^	*P* 4
		A∶A	C∶C	A∶C	C∶A		
II∶IV							
	nc2L1∶tnf223	138(1.13)	104(1.18)	66(0.81)	117(0.88)	0.19	0.046
III∶V							
	crc445∶tnc389	119(0.87)	93(0.84)	135(1.15)	146(1.14)	−0.15	0.041
V∶VI							
	tnc389∶nc6L13	23(0.68)	42(0.80)	66(1.19)	43(1.33)	−0.33	0.019
	crb115∶tnc088	96(0.84)	106(0.85)	148(1.14)	127(1.17)	−0.17	0.027
	crd176∶tnc088	100(0.85)	102(0.85)	151(1.13)	122(1.17)	−0.17	0.021
VI∶VII							
	nc6L13∶crf313	24(0.69)	39(0.79)	42(1.34)	65(1.19)	−0.34	0.026
	nc6L2∶crf313	22(0.69)	42(0.81)	38(1.35)	69(1.17)	−0.35	0.049

Six of the seven linkage groups of the *N. crassa* linkage maps incorporated markers involved in non-random associations with markers on different linkage groups.

1 A is NcA-Louisiana; C is NcC-India.

2 Expected haplotype counts are calculated from observed allele frequencies as follows: For a haplotype with allele i at locus 1 and allele j at locus 2, the expected genotypic count, N_ij_^expected^, is *p*_i_ *q*_j_N_total_, where *p* and *q* are allele frequencies at locus 1 and locus 2, respectively, and N_total_ is the number of progeny that were genotyped at loci 1 and 2.

3 D′ is D/D_max_, where D = *P*_11_ *P*_22_−*P*_12_ *P*_21_ and Dmax is *p*_1_ *q*_2_ or *p*_2_ *q*_1_, whichever is smaller; *P* is genotypic frequency, while *p* and *q* are allelic frequencies.

4 Unbiased *P* values for the Fisher exact test of genetic disequilibrium are estimated in using a Markov chain. *P* values reflect Bonferroni multiple test correction for the 21 nonidentical pairs of linkage groups.

Given partial intersterility between NcA-Louisiana and NcC-India and the segregation distortion that we observed, we predicted that observed significant non-random associations would be positive, indicating a deficit of recombinant haplotypes. However, only one significant marker pair (markers nc2L1 and tnf223 from LG II and IV, respectively) showed positive non-random associations (D' = 0.19). Surprisingly, the loci on the three other significant linkage-group pairs (III and V, V and VI, and VI and VII) showed negative, non-random associations, with D' values ranging from −0.35 to −0.15, implying overrepresentation of recombinant haplotypes. Overrepresentation of recombinant haplotypes could be evidence that an optimal *N. crassa* genome would include some mixture of clade-specific alleles. Indeed, a population genomic study of *N. crassa* NcA from the Caribbean Basin and the south-eastern United States identified a haplotype in the Louisiana population consisting of a four-gene “migrant tract” originating from an unidentified, genetically diverged population or species, concluding that this tract was fixed in the Louisiana population via a selective sweep [35]. Alternatively, if alleles are not fixed in the clades, overrepresented recombinant haplotypes could be analogous to selectively advantageous, intrapopulation haplotypes.

### Genetics of hybrid fruitbody abortion

**Figure 4:**
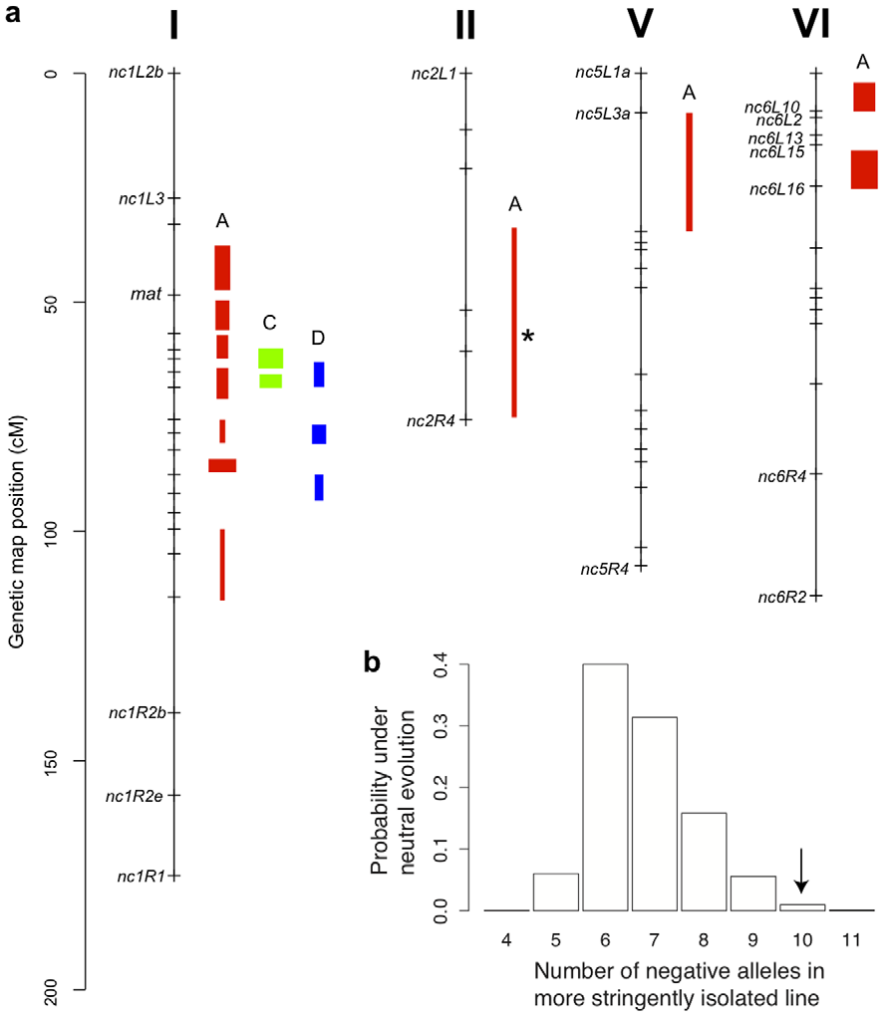
Genetics of hybrid fruitbody development in *N. crassa*. a. Chromosomes with QTLs are shown. Genome-anchored markers are labeled. Height of QTL rectangle indicates 1-LOD confidence interval; width is proportional to percent variance explained (range, 1.9%–11.0%). A = sympatric maternal; C = allopatric maternal; D = allopatric paternal; * = NcC-allele with positive effect. b. Rejection of neutral evolution for hybrid fruitbody abortion. The probability of observing a genetic architecture as or more biased toward negative alleles than NcC (arrow) is 0.0099. Allele bias was determined in 10,000 replicates of a neutral evolution model, assuming phenotypic disparities at least as great as in parental sympatric maternal fruitbody development, 11 QTLs, and genetic effects magnitudes distributed as in observed QTLs.

**Figure 5:**
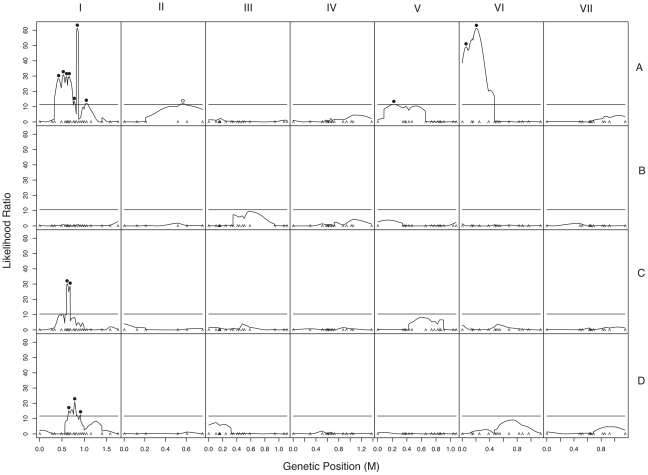
Composite interval mapping of hybrid fruitbody development in crosses between *N. crassa* and *N. intermedia*. The y-axes show the likelihood ratio statistic determined by composite interval mapping under the hypothesis that a QTL exists versus the null hypothesis that no QTL exists. The critical likelihood ratio threshold (horizontal line) reflects a Type I error of 0.05. The x-axis represents the seven linkage groups of the *N. crassa* genetic map (cM) generated in this study. Solid black circles indicate the positions of significant QTLs whose NcC alleles have negative effects on hybrid fruitbody development and open circles indicate QTLs whose NcC alleles have a positive effect on fruitbody development. Results for four traits are pictured: row 1, trait A—maternal influence on fruitbody development in *N. crassa* fertilized by sympatric *N. intermedia* from Tamil Nadu; row 2, trait B—paternal influence on fruitbody development by sympatric *N. intermedia* from Tamil Nadu fertilized by *N. crassa* from the mapping population; row 3, trait C—maternal influence on fruitbody development by *N. crassa* fertilized by allopatric *N. intermedia* from Africa; row 4, trait D—paternal influence on fruitbody development on allopatric *N. intermedia* from Africa fertilized by *N. crassa* from the mapping population.

We used composite interval mapping to identify QTLs for fruitbody development. The genetic basis of postmating reinforcement was revealed by mapping loci for maternal influence on sympatric fruitbody development (trait A, see Table 4). We identified 11 additive-effect QTLs for this trait (Figure 4a and Table 9; complete CIM scans for all traits are in Figure 5). Seven of the QTLs are located on LG I, including one of large effect, while LG II and V each contain a single broad QTL region of weak effect, and the left arm of LG VI harbors two other QTLs of large effect. The detected QTLs account for roughly 61% of trait variance, with the three loci of large effect accounting together for roughly 30% of trait variance. The inferred cytoplasm type (NcA-Louisiana or NcA-India) of the mapping strains did not significantly affect this trait.

**Table 9:** QTLs for hybrid fruitbody development in crosses between *N. crassa* and *N. intermedia*.

Trait	LG	Position (cM)	1-LOD Confidence Interval (cM)	Additive Effect	P.V.E.	LR
A) Female effect; sympatric (*n* = 493)						
	1	42.5	37.6–47.3	−0.5052	0.0621	28.3
	1	52.9	49.9–56.4	−0.5174	0.0596	30.9
	1	60.3	56.8–61.9^A^	−0.4912	0.0462	29.6
	1	66.3	64.3–71.1^BC^	−0.5098	0.0471	29.6
	1	77.6	75.6–80.7^D^	−0.4209	0.0223	13.4
	1	84.3	84.2–87.1	−0.6069	0.1102	61.3
	1	104.5	99.5–115.0	−0.3162	0.0186	12.2
	2	56.8	33.7–75.1	0.2543	0.0201	11.9
	5	22.1	8.6–34.5	−0.2896	0.0259	11.4
	6	5.5	2.0–8.3	−0.5333	0.0866	49.1
	6	20.6	16.8–25.2	−0.5928	0.1079	61.2
B) Male effect; sympatric (*n* = 345)						
			None detected			
C) Female effect; allopatric (*n* = 296)						
	1	61.4	60.4–64.8^AB^	−0.5116	0.0987	30.1
	1	68.3	65.3–68.3^C^	−0.4839	0.0892	28.6
D) Male effect; allopatric (*n* = 342)						
	1	65.3	63.0–68.5^BC^	−0.3516	0.0413	15.2
	1	78.5	76.7–80.9^D^	−0.4654	0.0565	21
	1	91.6	87.6–93.3	−0.4094	0.0341	12.5

The genetic architecture of hybrid fruitbody development in four types of *N. crassa* × *N. intermedia* crosses was investigated by composite interval mapping. The positions and effects of significant QTLs are listed in the table, and co-localization of QTLs for different cross types is noted.

LG is linkage group, LR is likelihood ratio, and P.V.E is percent variance explained. Additive effect is the effect of the NcC allele. One-LOD confidence intervals that share any superscripted letter code (^A,B,C,^ or ^D^) are overlapping. “Female effect” or “male effect” in the trait description indicates the role that members of the *N. crassa* mapping population played in the crosses.

For 10 of the 11 QTLs, the allele from the sympatric NcC-India parent has a negative effect on sympatric fruitbody development. Only the weak QTL on LG II has the opposite effect. The prevalence of negative alleles in the NcC-India background is consistent with evolution of abortion via directional natural selection. This inference can be statistically tested by the QTL sign test, which tests the null hypothesis that the observed genetic architecture was generated during neutral trait evolution, i.e., without selective advantage for negative alleles [32].

The QTL sign test assumes that all QTL effects are additive. We note that the accepted model of intrinsic, postzygotic isolation barriers involves negative among species-specific alleles in hybrids. However, our experiments were not designed to, nor can they, interrogate the genetics of hybrid dysfunction, but rather the genetics of evolutionary response to maladaptive hybridization. The genetic architecture of reinforcement may or may not involve epistatic effects. However, in contrast to the case of hybrid dysfunction, epistasis would involve within-genome, interaction effects among loci contributing to the reinforced barrier. To determine whether or not epistasis plays a role in the genetics of reinforced sympatric barriers in *N. crassa*, and to determine whether or not the genetic data conform to the assumptions of the QTL sign test, we performed a two-dimensional genome scan for interacting QTLs.

No significant interaction effect was detected. Moreover, the genome-wide maximum LOD score for any interaction effect was determined to be 16.7, well below the critical LOD score of 37.4 (for a Type I error of 0.05), which was estimated from 1000 permutations of the data. Because the two-way scan for genetic interactions among loci failed to find any significant or marginally significant interaction effects, the data are consistent with an additive genetic model and conform to the assumption of additivity required for the QTL sign test.

Given the observed number of positive and negative QTLs and the distribution of their effect magnitudes (gamma *_b_*_ = 0.034, *c* = 13.5_), and conditioned on the parental difference in fruitbody development after sympatric fertilization (F.D.S = 2), the null hypothesis of neutral trait evolution for abortion in NcC-India is rejected (QTL sign test, *P* = 0.0099) (Figure 4b). This result implies that fruitbody abortion in sympatric NcC-India–maternal×*N. intermedia*-paternal crosses (trait A) evolved under positive natural selection via a reinforcement mechanism.

One of the major QTLs on LG VI is flanked by two microsatellite loci, nc6L15 and nc6L16, and can therefore be physically located to a 135,874 bp region of the *N. crassa* genome containing 24 ORFs (Table S1) [36].

### Genetics of fruitbody development in the absence of reinforced barriers

We also investigated the genetics of fruitbody development in crosses not showing reinforced isolation (Traits B, C, and D, see Table 4). Note that NcC-Indian strains show enhanced isolation from Indian *N. intermedia* only when the NcC-Indian strain performs the maternal role [16]. We did not detect any QTLs for paternal influence on the development of sympatric fruitbodies (trait B), but in crosses to allopatric *N. intermedia* strains we detected two QTLs affecting maternal influence and three QTLs affecting paternal influence on fruitbody development (traits C and D, respectively; Figure 4a). All five of these loci are located on LG I. Four of the five allopatric QTLs co-localize with three of the sympatric QTLs on LG I, which could either indicate the presence of genes with pleiotropic effects or linked genes with trait-specific effects.

## Discussion

### Reinforcement alleles and segregation distortion on Linkage Group I

Linkage group I represents less than 24% of the genome, and it is striking that 75% of our QTLs map here. Interestingly, 73% of loci showing non-Mendelian segregation also map to linkage group I, so that every QTL on this linkage group is flanked by at least one marker showing segregation distortion. In all cases, the NcC alleles of the linkage group I QTLs have a negative effect on sympatric hybrid fruitbody development, and the NcC alleles of the linkage group I markers are underrepresented in the NcA×NcC *N. crassa* mapping population.

It is not known why one-fifth of genetic markers are distorted in favor of the NcA allele. It is possible that, because laboratory strains of *N. crassa* have historically been derived from the NcA clade, our crossing and progeny-isolation methods, which were developed for NcA-clade *N. crassa*, have inadvertently created a selective environment favoring NcA alleles at loci linked to distorted markers. It is also possible that distorter loci present in the NcA background are normally repressed through the action of NcA modifier loci, but become unrepressed and active in some recombinant NcA×NcC genotypes. Although segregation distortion can be caused by nuclear-cytoplasm incompatibilities, it is unlikely to be the cause in this case. The *Neurospora* mapping population comprises a mixture of individuals with NcA-Louisiana cytoplasm and NcC-India cytoplasm. Moreover, even in individuals with NcC-India cytoplasm, linkage group I markers are distorted in favor of the NcA allele, with an overall NcA allele frequency of 0.61 for markers on this linkage group.

Another hypothesis is that reinforcement alleles themselves can pleiotropically cause ascospore inviability in conspecific, inter-clade crosses. Laboratory crosses between members of the NcA and NcC clades are partially intersterile, usually resulting in <50% ascospore viability [15]. In *Neurospora*, all products of meiosis contribute to the ascospore cohort, so segregation distortion most likely results from inviability of hybrid ascospores carrying the disfavored NcC allele(s).

Pleiotropy for reinforcement and reproductive isolation between allopatric conspecifics has previously been observed in animals [37], [38]. Reduced conspecific fertility can present a challenge to the evolution of reinforced barriers, since the fitness advantage of avoiding hybridization must outweigh the cost of lower conspecific fertility. However, restricted migration between conspecific populations should reduce the incidence of interpopulation mating and reduce the fitness costs associated with pleiotropic effects on conspecific fertility. The NcC and NcA clades are geographically separated, so limited interclade migration would reduce the fitness cost to NcC of lower fertility with NcA and facilitate the spread of reinforcement alleles in the NcC clade. If the pleiotropy hypothesis is correct, the evolution of reinforcement QTL on linkage group I could be a partial explanation for the evolution of incomplete reproductive isolation between the NcC and NcA clades.

Notably, QTLs on other linkage groups (II, V and VI) are flanked by markers showing Mendelian segregation, so that for these QTLs there is no suggestion of pleiotropic negative effects on within-species, inter-clade reproduction. Moreover, the two reinforcement QTLs on linkage group VI lie in the vicinity of three markers (tnc088, nc6L13, nc6L2), which participate in non-random associations with loci on other linkage groups, such that recombinant, non-parental haplotypes are overrepresented in the mapping population. Therefore the patterns of marker inheritance near QTLs on these other linkage groups do not suggest any connection between reinforcement QTLs and isolation of conspecific allopatric *N. crassa* populations.

We note that linkage group I contains the mating-type locus of *Neurospora*, and that some studies have found that reproductive isolation loci are more prevalent on sex-determining chromosomes than on autosomes [39], [40]. It is true that in a closely related species, *N. tetrasperma*, recombination is suppressed over a large region of Linkage Group I in a process considered analogous to an early stage of sex-chromosome evolution [41]. However, no recombination block exists on linkage group I of *N. crassa*. Additionally, *Neurospora* species are hermaphroditic, so the mating-type locus determines mating compatibility, rather than sexual role. We therefore consider it unlikely that the same forces that cause reproductive isolation loci to preferentially accumulate on sex chromosomes can account for the prevalence of the observed QTLs on linkage group I.

### A previously identified reproductive isolation QTL on linkage group I

Earlier genetic studies of reproductive isolation in *Neurospora* identified a QTL on linkage group I as the *N. crassa* member of a Dobzhansky-Muller incompatibility locus-pair responsible for a severe defect in hybrid perithecial development between allopatric *N. crassa* and *N. intermedia* when *N. crassa* acts as the male partner [42], [43]. These incompatibility loci were first identified in populations of *N. crassa*×*N. intermedia* hybrids evolved under divergent environmental conditions in a test of the hypothesis that ecological adaptation can incidentally drive reproductive isolation [42]. Subsequent mapping determined that the incompatibility was caused by interactions between an *N. crassa* locus (*dma* on linkage group I) and an *N. intermedia* locus (*dfe* on linkage group V) [43]. Considering that both the geographic relationship of the species and gender role of *N. crassa* differ between this study and ours, it is very interesting that the *N. crassa dma* locus maps to a region of linkage group I that potentially coincides with the locations of QTLs identified in our study. Direct comparison between mapping results is prevented by the absence of sequence anchored, microsatellite markers in this region of our map.

### Conclusions

Sexual microbes are likely to have simple premating recognition mechanisms, but will nevertheless experience selective pressure to avoid maladaptive hybridization. When evolution of premating barriers is constrained, microbial reinforcement may be more likely to involve non-premating-recognition mechanisms, including differentiated substrate or host fidelities [10] or evolution of divergent mating kinetics [44]. Here we have shown that selective abortion of hybrid fruitbodies by *N. crassa* fertilized by sympatric *N. intermedia* had the potential to evolve by natural selection by demonstrating that maternal colonies that abort hybrid fruitbodies are subsequently able to mate normally with conspecifics and can have higher reproductive fitness. We then show that the genetic architecture of hybrid fruitbody abortion is consistent with evolution via directional natural selection. Plants and animals are known to sometimes selectively abort otherwise viable embryos, thereby restricting parental investment to offspring with higher potential fitness [45]. Our data show that microbes like *Neurospora*, which provide costly parental care and are capable of multiple matings, are capable of undergoing reinforcement selection for selective abortion of hybrid offspring. Further studies on the evolution and genetics of reproductive isolation in microbial eukaryotes will be needed to challenge this hypothesis.

## Materials and Methods

### Neurospora strains and culture conditions

The biology of *Neurospora*, the evolutionary relationships among species and clades, and the biogeography of reproductive isolation between *N. crassa* and *N. intermedia* have been described previously [15], [16]. Culturing, crossing, and isolation of ascospore progeny were performed as previously described [15], [17]. Table S1 lists the wild-collected *Neurospora* strains used in this study. The QTL mapping population created for this study has been deposited with the Fungal Genetics Stock Center, Kansas City, Missouri.

### Sequential fertilization

Sequential fertilization was performed according to the methods of Howe and Prakash [27], except that at the first mating time point (5 days after inoculation of the NcC-India maternal strain), the conidial suspension of one fertilizing strain (either an NcC-India strain as conspecific positive control; an allopatric *N. intermedia* (African (*n* = 2) or Caribbean (*n* = 2)); sympatric *N. intermedia* (Indian (*n* = 2)); or water negative control) was applied to 50% of the plate, while at the second time point (10 days after maternal inoculation), the fertilizing suspension of NcC-India was applied to two 1 cm^2^ spots located 1.5 cm–2 cm from the edge of the first fertilization. Three replicates were performed for a total of 24 plates. Fertility was scored 20 days after maternal inoculation using a 0–6 reproductive success scale (RSS) [15]. The effects of cross type and geographic origin and strain identity of the first–time-point fertilizing males on reproductive success of the second–time-point crosses were analyzed using a semi-parametric, proportional hazards model, with nested effects, as implemented by JMP 5.0.1a.

### Genotyping

We obtained genomic DNA for each member of the QTL mapping population following the protocol of Dettman et al. [15]. AFLP and microsatellite primer sequences are shown in Table 5, Table 6, and Table 7. The *mat-a1* and *mat-A1* loci were amplified by multiplex PCR with the following primers: Ba1-5, AAGAAGAAGGTCAACGGCTTCATG; Ba1-3, CCAGAGCCATGTTCTAGGAATCATT; Sa1-5, CGTCGATGGCAATCTTTTCTGGAA; and Sa1-3, ATTGGCATCGTAGTTGAGAAGCTT [46]. The *mat-a1* and *mat-A1* fragments were distinguished by agarose gel electrophoresis. Genomic DNA was prepared for amplification of AFLP loci with the Invitrogen AFLP Core Reagent Kit. Selective “E” primers were 5′-modified with either 6-FAM or HEX fluorescent dye, and products of selective amplification were electrophoretically separated on an ABI 3100 genetic analyzer, and data were collected and analyzed with the ABI software GeneScan and Genotyper (Applied Biosystems, Inc., Carlsbad, CA).

Microsatellite loci in targeted genomic regions (e.g., chromosome ends and QTL regions) were selected from a published list of SSR in *N. crassa* [47], and primers were designed with Primer3 on the web [48]. Forward primers were 5′-fluorescently labeled with NED, 6-FAM, or HEX dyes, and size data were collected as for AFLP markers, above.

### Linkage analysis

Linkage analysis was performed with MAPMAKER/EXP 3.0 [49]. Loci were sorted into sets of linked markers iteratively at a linkage threshold of 3.0 LOD and 50 cM (Kosambi) and then 6.0 LOD and 30 cM using the “group” command. For each linkage group, the “order” command was used to identify the best marker order with a window of 7 markers and a log-likelihood exclusion threshold of 2.0, followed by attempted placement of excluded markers at log-likelihood 1.0. Marker order was confirmed using the “ripple” command to permute five neighboring loci at a time and flag any alternative orders within a log-likelihood of 2.0 of the best order. Markers that could not be placed confidently according to these criteria were discarded. Linkage groups were assigned to the seven chromosomes of *N. crassa* based on inclusion of multiple microsatellite markers targeted to that chromosome.

Mendelian segregation of markers was checked in R/qtl [50] with the “geno.table” command. Linkage disequilibrium in pairs of physically unlinked markers was tested in Genepop 3.4 [51], using option 2, which uses a Fisher exact test implementing a Markov chain to estimate an unbiased *P*-value. Experiment wide significance threshold (Type I error α = 0.05) was determined by Bonferroni correction for the 21 non-identical linkage-group pairs.

### QTL analysis

We investigated the genetics of hybrid fruitbody development in four kinds of *N. crassa*×*N. intermedia* matings (Table 4). Crosses were performed on synthetic cross media in 16×100 mm tubes. At 10 days post-fertilization, fruitbody development scores (FDS) were recorded following a four-category scale: 0, no fruitbody development; 1, early abortion (small fruitbodies without apical pores (ostioles)); 2, late abortion (larger fruitbodies with ostioles, but lacking “beaks”); and 3, fully developed (large flask-shaped fruitbodies with ostioles and “beaks”). Some crosses resulted in a mixture of two consecutive fruitbody development stages and were recorded as half steps in the scale (e.g., crosses with early- and late-aborted fruitbodies were recorded as 1.5).

Quantitative trait loci (QTLs) were identified by composite interval mapping (CIM) using Windows QTL Cartographer, v2.5 [52]. CIM was performed under model 6 with 5 control markers and a window size of 20 cM using a 1 cM walk speed. At each step the likelihood ratio statistic (LR) testing the hypothesis that a QTL exists versus the null hypothesis that no QTL exists was determined. For each trait, a critical LR threshold reflecting a Type I error of 0.05 was estimated by permuting the data 1000 times. Significant QTLs were CIM maxima whose LR exceeded the critical threshold and whose 95% confidence intervals were discontinuous with those of other CIM maxima. Ninety-five percent support intervals were estimated as the area bounded by 1-LOD drops in the LR where LOD = log_10_(LR/2 ln 10).

The null hypothesis of neutral trait evolution for sympatric hybrid fruitbody abortion (the reinforcement trait) was tested by subjecting the genetic effects data to a QTL sign test [32], as implemented by the QTLsigntest software provided by H. A. Orr. Because the QTL sign test assumes an additive genetic model, we first scanned for epistatic loci using “scantwo” of R/qtl using the expectation-maximization, interval mapping algorithm and multipoint genotype probabilities calculated using the “calc.genoprob” command with a step size of 2.5 and an error probability of 0.01. For each chromosome position the likelihood ratio statistic comparing the full epistatic model to the two-locus additive model was determined For each trait, a critical likelihood ratio statistic threshold reflecting a Type I error of 0.05 was estimated by permuting the data set 1000 times.

QTLsigntest determines how likely the proportion of loci with positive vs. negative additive effects is under a neutral model of complex trait evolution, when conditioned on the magnitude of the trait difference in the parent strains, the number of detected QTLs, the threshold of detection, and distribution of the absolute value of additive effects, which are all empirically determined. QTLsigntest was parameterized as follows: Parental RSS difference = 2; number of QTLs = 11; detection threshold = 0.25; effects distribution gamma (shape = 13.5, scale = 0.034).

## Supporting Information

Table S1 Candidate genes for postmating reinforcement in *N. crassa*. The ORFs found between microsatellite markers nc6L15 and nc6L16 on linkage group VI, which flank a major female-fertility QTL affecting sympatric hybrid fruitbody development, are listed. The presence of these ORFs in publicly available reproductive EST libraries is noted. The Sexual, Perithecial, Sperithecial, and Westergaard EST libraries were constructed from cDNA harvested from mycelia undergoing sexual development. All data are from the Broad Institute *Neurospora crassa* database.
(DOC)
