# Supplementary material for: Genetic Architecture of a Reinforced, Postmating, Reproductive Isolation Barrier between Neurospora Species Indicates Evolution via Natural Selection
Source: PLoS Genet. 2011 Aug 18;7(8):e1002204. doi: 10.1371/journal.pgen.1002204 (PMC3158040; doi:10.1371/journal.pgen.1002204)
Supplement: Table S1 — Candidate genes for postmating reinforcement in N. crassa. The ORFs found between microsatellite markers nc6L15 and nc6L16 on linkage group VI, which flank a major female-fertility QTL affecting sympatric hybrid fruitbody development, are listed. The presence of these ORFs in publicly available reproductive EST libraries is noted. The Sexual, Perithecial, Sperithecial, and Westergaard EST libraries were constructed from cDNA harvested from mycelia undergoing sexual development. All data are from the Broad Institute Neurospora crassa database. (DOC) [file pgen.1002204.s001.doc]

Table S1. Candidate genes for postmating reinforcement in *N. crassa*.

| Locus | Gene Name; Description (Symbol) | Presence in Reproductive EST Libraries  (No. of clones) |
| --- | --- | --- |
| NCU12117.4 | Hypothetical protein |  |
| NCU04718.4 | Hypothetical protein; Fungal protein of unknown function | Sexual (1) |
| NCU04719.4 | Hypothetical protein |  |
| NCU04720.4 | Nitrite reductase (nit-6) | Westergaard (3) |
| NCU04721.4 | Neutral ceramidase |  |
| NCU04722.4 | Hypothetical protein |  |
| NCU04723.4 | Hypothetical protein |  |
| NCU04724.4 | VHS domain-containing protein |  |
| NCU04725.4 | N-acetylglucosamine-6-phosphate deacetylase | Sexual (1) |
| NCU04726.4 | beta-N-acetylglucosaminidase |  |
| NCU04727.4 | Glucosamine-6-phosphate deaminase |  |
| NCU04728.4 | Hexokinase-1 |  |
| NCU04729.4 | Hypothetical protein; Similar to *vib-1* |  |
| NCU04730.4 | Post-transcriptional silencing protein (qde-2) | Sperithecial (1) |
| NCU04731.4 | HLH transcription factor (sah-2) |  |
| NCU04732.4 | Hypothetical protein | Perithecial (3), Sexual (2), Sperithecial (2) |
| NCU04733.4 | UvrD/REP helicase (mus-50) |  |
| NCU04734.4 | Hypothetical protein; Heterokaryon incompatibility protein (HET) |  |
| NCU04735.4 | Hypothetical protein |  |
| NCU12118.4 | Hypothetical protein |  |
| NCU04736.4 | Plasma membrane calcium-transporter ATPase (nca-2) |  |
| NCU04737.4 | Chromatin regulatory protein; Histone deacetylase (sir2) |  |
| NCU04738.4 | Hypothetical protein; Primase zinc finger and oligonucleotide binding fold domains | Sexual (1) |
| NCU04740.4 | Hypothetical protein; RNase H2 complex component domain |  |
